# Design, Conduct, and Analysis of Externally Controlled Trials

**DOI:** 10.1001/jamanetworkopen.2025.30277

**Published:** 2025-09-04

**Authors:** Jiali Liu, Minghong Yao, Mingqi Wang, Wan Jie, Yanmei Liu, Xiaochao Luo, Jiayidaer Huan, Kelin Deng, Ke Deng, Kang Zou, Ying Zhang, Ling Li, Xin Sun

**Affiliations:** 1 Institute of Integrated Traditional Chinese and Western Medicine and Chinese Evidence-Based Medicine Center and Cochrane China, West China Hospital, Sichuan University, Chengdu, China; 2 Department of Epidemiology and Biostatistics, West China School of Public Health and West China Fourth Hospital, Sichuan University, Chengdu, China; 3 Department of Oncology, Guang’anmen Hospital, China Academy of Chinese Medical Sciences, Beijing, China; 4 Department of Health Research Methods, Evidence, and Impact, McMaster University, Hamilton, Ontario, Canada

## Abstract

**Importance:**

Externally controlled trials (ECTs) can serve as an alternative in settings where randomized clinical trials (RCTs) are unfeasible. However, the methodological rigor of ECTs, particularly with regard to bias control, is often inadequately assessed, which can compromise the validity of studies and lead to incorrect decisions.

**Objective:**

To examine the design, conduct, and analysis characteristics of current ECTs and to assess whether appropriate methods were used to control bias.

**Design, Setting, and Participants:**

This cross-sectional study searched PubMed for ECTs published between January 1, 2010, and December 31, 2023. Eligible ECTs included single-arm trials with an external control or that used a treatment group from an RCT compared with an external control and evaluated the efficacy and/or safety of a drug or medical device. Data analysis was conducted from March 5 to 20, 2025.

**Main Outcomes and Measures:**

Extracted information included design characteristics, external control data sources, transparency in covariate selection, statistical methods, and the use of sensitivity and quantitative bias analyses. The characteristics of included ECTs were compared between journals in the top 25% in their Journal Citation Reports category (Q1) and non-Q1.

**Results:**

This study included 180 ECTs, of which 85 (47.2%) focused on oncology. Only 64 (35.6%) provided reasons for using external controls, and 29 (16.1%) were prespecified to use external controls. The main sources of external controls were clinical (also termed real-world) data (98 [54.4%]) and trial-derived controls (67 [37.2%]), while concurrent data collection with the treatment arm was relatively infrequent (18 [10.0%]). Only 14 studies (7.8%) conducted feasibility assessments to evaluate the adequacy of data sources, and 13 (7.2%) specified how to handle missing data in external control datasets. Covariate selection procedures were described in 37 of the 164 studies (22.6%) that reported important covariates. Sixty studies (33.3%) used statistical methods to adjust for important covariates when generating the external control, with the propensity score method being the most common (35 of 60 [58.3%]). Among 120 ECTs that generated external controls without statistical methods, 91 (75.8%) used univariate analysis to estimate treatment effects, and only 18 (15.0%) used multivariable regression analysis. Sensitivity analyses for primary outcomes were performed in 32 studies (17.8%), and quantitative bias analyses (2 [1.1%]) were nearly absent. ECTs in Q1 journals were more likely to prespecify the use of external controls (χ^2^_1_ = 9.86; *P* = .002) and provided rationales for using external controls (χ^2^_1_ =  4.33; *P* = .04). Thirteen recommendations for the careful practice of ECTs are proposed.

**Conclusions and Relevance:**

In this cross-sectional study of ECTs, current practices in the design, conduct, and analysis were suboptimal, limiting their reliability and credibility. The study identified several critical methodological issues, such as the lack of justification for using external controls, failure to prespecify external controls in the protocol, insufficient use of confounding adjustment techniques, inadequate sensitivity analyses, and almost complete absence of quantitative bias analyses. Therefore, actionable suggestions for future ECT practices are proposed.

## Key Points

**Question:**

How are externally controlled trials (ECTs) currently designed, conducted, and analyzed in practice, and do they use methods, such as statistical methods, to control bias?

**Findings:**

In this cross-sectional analysis of 180 ECTs published between 2010 and 2023, several critical methodological issues were identified, and actionable recommendations were proposed to support improvements in future ECT practices.

**Meaning:**

These findings emphasize the need for researchers to improve ECT practices and highlight the urgent need for collaboration among researchers, regulators, and journal editors to develop standardized methodological and reporting guideline for ECTs.

## Teaser

This cross-sectional study analyzes published externally controlled trials to assess the diversity of study designs, sources and characteristics of external control data, statistical methods used for confounding adjustment, and the application of quantitative bias analysis.

## Introduction

Randomized clinical trials (RCTs) are considered the gold standard for generating clinical evidence of the efficacy and safety of medical interventions. However, in some scenarios, such as rare diseases, it may not be feasible to conduct RCTs.^1^ In such cases, single-arm trials are sometimes used as pivotal trials. However, because there is no comparator treatment, the benefit of the new treatment over existing treatments remains unconfirmed, reducing the level of confidence compared with RCTs.^2^ The US Food and Drug Administration (FDA) guidance documents acknowledge that the use of external controls is acceptable when internal controls are not feasible or ethical in certain circumstances.^3,4^ According to the international Conference on Harmonization E10 guidance, “an externally controlled trial (ECT) compares a group of subjects receiving the test treatment with a group of patients external to the study, rather than to an internal control group consisting of patients from the same population assigned to a different treatment.”^5^ Recently, evidence generated from ECTs has increasingly been used to support successful regulatory submissions and health technology assessments (HTAs).^6,7,8^

However, conducting robust and rigorous ECTs is challenging.^9,10^ Without randomization, a major concern in ECTs is that patient attributes influencing outcomes may differ between the external control and the treatment arm. This would introduce biases such as confounding, selection bias, and survivor–lead-time bias, risking study validity and potentially leading to incorrect decisions.^6,11^ The FDA has recognized these issues and made some recommendations to sponsors and investigators considering the use of ECTs.^12^ Some researchers have provided key methodological considerations for the design of ECTs, such as conducting feasibility assessment to determine the adequacy of available data sources to serve as external control arms, using appropriate statistical methods to adjust for baseline imbalances between the treatment and external control arms.^2,13,14^ Despite these efforts, in practice it can be unclear how current ECTs are designed, conducted, and analyzed, and whether they use statistical methods to control bias.

We therefore conducted a cross-sectional study of ECTs published since 2010 to assess the diversity of study designs, sources, and characteristics of external control data, statistical methods used for confounding adjustment, and the application of quantitative bias analysis. Based on our findings, we provide recommendations to promote best practices in ECT design and analysis.

## Methods

The protocol was registered online at Open Science Framework registries.^15^ This was a cross-sectional study that adopted a literature survey approach to describe and analyze the characteristics of included studies. As the unit of analysis consisted of published studies, ethics approval and informed consent were not applicable. We followed the Strengthening the Reporting of Observational Studies in Epidemiology (STROBE) reporting guideline.

### Data Sources

In January 2025, we searched PubMed for ECTs published between January 1, 2010, and December 31, 2023, without language restrictions. We used Medical Subject Headings and free text terms associated with single-arm trial, clinical trial, and external control to search for potentially eligible studies (eAppendix in Supplement 1).

### Eligibility Criteria and Study Process

We included a study if it was a single-arm trial with an external control (eg, historical control) or if it used a treatment group from an RCT compared with an external control and if it aimed to evaluate the efficacy and/or safety of a drug or medical device involving human participants. We excluded studies that used summary-level estimates as the external control and those reported as pilot studies, protocols, research letters, conference abstracts, and short reports. We included ECTs reported as both primary and nonprimary analyses (eg, exploratory or secondary analyses) to provide a more comprehensive overview of the current methodological practices.

Four pairs of investigators (M.W. and W.J.; Y.L. and X.L.; J.H. and Kelin Deng; Ke Deng and K.Z.) trained in research methods independently and in duplicate used pilot-tested and standardized forms to screen titles, abstracts, and full texts for eligibility and to extract data from included ECTs. All trials that met the inclusion criteria following the screening process were included in the analysis, with no sample size restrictions. Disagreements that arose during this process were resolved by discussion or consultation with a third researcher (J.L. or L.L.).

### Data Extraction

We developed a data extraction form to collect data from eligible ECTs. The initial version was formulated by 2 investigators (J.L. and M.Y.). We then piloted the data extraction form by collecting data from 30 ECTs and holding a meeting to discuss the suitability and practicality of the listed items. After further refinements, we conducted a second pilot and settled discrepancies through group discussion. Based on the piloted and revised data extraction form, we collected the following information on each included ECT: the characteristics of the external control data sources used, the methodology used to generate the external controls, the methodology used for the statistical analyses to estimate the treatment effects, and the methodology used for the sensitivity and quantitative bias analyses (eMethods 1 and 2 in Supplement 1).

### Statistical Analysis

We conducted descriptive analyses of general and methodological characteristics of the included studies. For all descriptive analyses, frequencies and percentages were used to summarize dichotomous variables, and mean and SD or median and IQR were used for continuous variables. Journals were classified as within the top 25% in their Journal Citation Reports category (Q1) or non-Q1 based on their 2024 impact factor ranking. To compare the characteristics of included trials published in Q1 and non-Q1 journals, we used the χ^2^ test or Fisher exact test for dichotomous variables, independent samples *t* test for continuous variables when the distribution was normal, or the Mann-Whitney test otherwise. We used R software, version 4.3.2 (R Program for Statistical Computing) for data analysis. A 2-sided *P* ≤ .05 was considered statistically significant. Data analysis was conducted from March 5 to 20, 2025.

## Results

Of 1491 potentially relevant reports, 642 were retrieved for full-text screening, then 180 ECTs published in 178 reports were found to be eligible.^16,17,18,19,20,21,22,23,24,25,26,27,28,29,30,31,32,33,34,35,36,37,38,39,40,41,42,43,44,45,46,47,48,49,50,51,52,53,54,55,56,57,58,59,60,61,62,63,64,65,66,67,68,69,70,71,72,73,74,75,76,77,78,79,80,81,82,83,84,85,86,87,88,89,90,91,92,93,94,95,96,97,98,99,100,101,102,103,104,105,106,107,108,109,110,111,112,113,114,115,116,117,118,119,120,121,122,123,124,125,126,127,128,129,130,131,132,133,134,135,136,137,138,139,140,141,142,143,144,145,146,147,148,149,150,151,152,153,154,155,156,157,158,159,160,161,162,163,164,165,166,167,168,169,170,171,172,173,174,175,176,177,178,179,180,181,182,183,184,185,186,187,188,189,190,191,192,193^ Of these, 98 reports were published in Q1 journals and 82 in non-Q1 journals (eFigure 1 in Supplement 1).

### General Characteristics of ECTs

Table 1 shows the general characteristics of the included ECTs. Of the 180 ECTs, 165 (91.7%) were single-arm trials compared with external controls and 123 (68.3%) clearly specified trial registration. Almost half of ECTs (85 [47.2%]) were reported in oncology, 151 (83.9%) focused on drugs, and 29 (16.1%) involved medical devices. One hundred and sixty-four ECTs (91.1%) used 1 external control as a comparator and the most commonly used type of external control was standard care (62 [34.4%]). Only 29 ECTs (16.1%) were prespecified to use an external control in the protocol, 64 (35.6%) provided reasons for using external controls, of which 26 (40.6%) highlighted the challenge of recruiting enough patients for rare diseases. Among 180 ECTs, 105 (58.3%) were classified as primary analyses, with 21 (20.0%) of these prespecified in the protocol to use an external control; 75 analyses (41.7%) were classified as nonprimary, of which 8 (10.7%) prespecified the use of an external control (eTable 1 in Supplement 1). Most of the studies received financial support, and 86 (47.8%) declared conflict of interest for some authors. Few ECTs (19 [10.6%]) calculated the sample size for the external control arm (eTable 2 in Supplement 1).

**Table 1.** General Characteristics of Included Studies

Characteristic	Study group, No. (%)			*P* value^a^	χ^2^ Value^b^
	Total (N = 180)	Q1 journals (n = 98)	Non-Q1 journals (n = 82)		
Type of study					
Single-arm trial vs external control	165 (91.7)	90 (91.8)	75 (91.5)	>.99	0
An arm derived from RCT vs external control	15 (8.3)	8 (8.2)	7 (8.5)		
Analysis type of ECTs as reported					
ECTs classified as primary analyses	105 (58.3)	53 (54.1)	52 (63.4)	.27	1.24
ECTs classified as nonprimary analyses	75 (41.7)	45 (45.9)	30 (36.6)		
Medical area					
Oncology	85 (47.2)	48 (49.0)	37 (45.1)	.71	0.13
Nononcology	95 (52.8)	50 (51.0)	45 (54.9)		
Type of treatment					
Drug	151 (83.9)	86 (87.8)	65 (79.3)	.18	1.79
Medical device	29 (16.1)	12 (12.2)	17 (20.7)		
No. of external controls					
1	164 (91.1)	86 (87.8)	78 (95.1)	.14	2.15
≥2	16 (8.9)	12 (12.2)	4 (4.9)		
Type of external control					
Drug	47 (26.1)	27 (27.6)	20 (24.4)	.77^c^	NA
Device	16 (8.9)	7 (7.1)	9 (11.0)		
Placebo	5 (2.8)	3 (3.1)	2 (2.4)		
Standard care	62 (34.4)	35 (35.7)	27 (32.9)		
No treatment	48 (26.7)	24 (24.5)	24 (29.3)		
Not specified	2 (1.1)	2 (2.0)	0		
Reason for using external control reported	64 (35.6)	42 (42.9)	22 (26.8)		
Rare diseases (not easy to recruit patients)	26 (40.6)	23 (54.8)	3 (13.6)	.04	4.33
Placebo control is difficult to implement	16 (25.0)	7 (16.7)	9 (40.9)		
Accelerate product development	6 (9.4)	3 (7.1)	3 (13.6)		
Ethical considerations	18 (28.1)	11 (26.2)	7 (31.8)		
Recommended by guidelines	7 (10.9)	2 (4.8)	5 (22.7)		
No efficacious treatments	3 (4.7)	3 (7.1)	0		
Other^d^	17 (26.6)	12 (28.6)	5 (22.7)		
Trial registration	123 (68.3)	74 (75.5)	49 (59.8)	.04	4.42
Trial protocol available	125 (69.4)	75 (76.5)	50 (61.0)	.04	4.38
Prespecified for using external control^e^	29 (16.1)	24 (24.5)	5 (6.1)	.002	9.86
Source of funding					
Private for profit	79 (43.9)	44 (44.9)	35 (42.7)	.88	0.02
Private or public not for profit	49 (27.2)	28 (28.6)	21 (25.6)	.78	0.08
Government	40 (22.2)	27 (27.6)	13 (15.9)	.09	2.89
No funding	22 (12.2)	11 (11.2)	11 (13.4)	.83	0.05
Not specified	16 (8.9)	8 (8.2)	8 (9.8)	.91	0.01
Conflict of interest disclosure					
The authors declared no conflict of interest	83 (46.1)	40 (40.8)	43 (52.4)	.18	3.46^f^
The authors declared conflict of interest for some authors	86 (47.8)	53 (54.1)	33 (40.2)		
Not specified	11 (6.1)	5 (5.1)	6 (7.3)		

Abbreviations: ECT, external controlled trial; NA, not applicable; Q1, journals in the top 25% in their Journal Citation Reports category; RCT, randomized clinical trial.

^a^ Unless otherwise indicated, calculated using the Pearson χ^2^ test.

^b^ Unless otherwise indicated, *df* = 1.

^c^ Calculated using the Fisher exact test.

^d^ Other reasons include selecting an external control group simply due to the lack of a control in a single-arm trial or choosing clinical data as the external control to explore the actual efficacy of the treatment in a clinical setting.

^e^ Indicates an external control that was prespecified during the protocol stage.

^f^ *df* = 2.

Compared with ECTs published in non-Q1 journals, those published in Q1 journals were more likely to provide a rationale for the use of external control (42 of 98 [42.9%] vs 22 of 82 [26.8%]; χ^2^_1_ = 4.33, *P* = .04), perform trial registration (74 of 98 [75.5%] vs 49 of 82 [59.8%]; χ^2^_1_ = 4.42, *P* = .04), provide a trial protocol (75 of 98 [76.5%] vs 50 of 82 [61.0%]; χ^2^_1_ = 4.38; *P* = .04), and prespecify external controls as comparator in the study protocol (24 of 98 [24.5%] vs 50 of 82 [61.0%]; χ^2^_1_ = 9.86, *P* = .002).

### Characteristics of External Control Data Sources

Table 2 shows the characteristics of the external control data sources. Of the 180 ECTs, 98 (54.4%) external control data sources were derived from clinical (also termed real-world) data and 67 (37.2%) were derived from trial data. Only 18 ECTs (10.0%) involved contemporaneous data collection compared with the treatment arm (compared with 162 [90.0%] using noncontemporaneous historical data). For partially concurrent and nonconcurrent trials, the median time since external data collection was approximately the last 5 (IQR, 2-9) years. Only 14 ECTs (7.8%) preliminarily assessed the external control data, of which 8 (57.1%) and 10 (71.4%) assessed accuracy and completeness, respectively. Of 180 ECTs, 117 (65.0%) included patient diagnostic criteria, 112 (62.2%) used inclusion criteria, and 96 (53.3%) used exclusion criteria that were the same as or similar to those in the treatment arm. One hundred and eight studies (60.0%) reported treatment characteristics of the external control, 40 (22.2%) specified combined interventions, and 30 (16.7%) reported treatment adherence. Most studies (168 [93.3%]) used the same or similar definitions of outcome measures. Only 46 studies (25.6%) predefined the index date, of which 39 (84.8%) were similar to those in the treatment arm. Very few ECTs (13 [7.2%]) specified how to handle missing data when obtaining external control data. No significant differences were observed between Q1 and non-Q1 journals, except for the diagnostic criteria used between the treatment and external controls arm (χ^2^_1_ = 6.25; *P* = .04).

**Table 2.** Characteristics of External Control Data Sources

Characteristic	Study group. No. (%)			*P* value^a^	χ^2^ Value^b^
	Total (N = 180)	Q1 journals (n = 98)	Non-Q1 journals (n = 82)		
Sources of external control data					
Clinical data	98 (54.4)	52 (53.1)	46 (56.1)	.98^c^	NA
Trial data	67 (37.2)	37 (37.8)	30 (36.6)		
Clinical and trial data	1 (0.6)	1 (1.0)	0)		
Not specified	14 (7.8)	8 (8.2)	6 (7.3)		
Time periods of external data collection compared with treatment arm					
Complete overlapping of time periods (concurrent)	18 (10.0)	7 (7.1)	11 (13.4)	.32	3.50^d^
Time periods with partial overlap (partially concurrent)	27 (15.0)	14 (14.3)	13 (15.9)		
No overlap in time periods (nonconcurrent)	91 (50.6)	55 (56.1)	36 (43.9)		
Not specified	44 (24.4)	22 (22.4)	22 (26.8)		
For partial and noncontemporaneous studies, difference in data collection time between the treatment and external control arms, median (IQR), y^e^	5 (2-9)	5 (2-10)	3.5 (1.75-7.25)	.06^f^	NA
Preliminary assessment of external data	14 (7.8)	9 (9.2)	5 (6.1)	.62	0.24
Assessing the accuracy of external data	8 (57.1)	4 (44.4)	4 (80.0)	.30^c^	
Assessing the completeness of external data	10 (71.4)	8 (88.9)	2 (40.0)	.10^c^	
Diagnostic criteria (compared with treatment arm)					
Same or similar criteria	117 (65.0)	56 (57.1)	61 (74.4)	.04	6.25^g^
Different criteria	12 (6.7)	7 (7.1)	5 (6.1)		
Not specified	51 (28.3)	35 (35.7)	16 (19.5)		
Inclusion criteria (compared with treatment arm)					
Same or similar criteria	112 (62.2)	57 (58.2)	55 (67.1)	.33	2.21^g^
Different criteria	19 (10.6)	13 (13.3)	6 (7.3)		
Not specified	49 (27.2)	28 (28.6)	21 (25.6)		
Exclusion criteria (compared with treatment arm)					
Same or similar criteria	96 (53.3)	49 (50.0)	47 (57.3)	.60	1.03^g^
Different criteria	18 (10.0)	11 (11.2)	7 (8.5)		
Not specified	66 (36.7)	38 (38.8)	28 (34.1)		
Treatment characteristics reported	108 (60.0)	54 (55.1)	54 (65.9)	.19	1.73
Combined interventions reported	40 (22.2)	20 (20.4)	20 (24.4)	.65	0.21
Treatment adherence reported	30 (16.7)	17 (17.3)	13 (15.9)	.95	0.004
Definition of outcomes (compared with treatment arm)					
Same or similar criteria	168 (93.3)	93 (94.9)	75 (91.5)	.76^c^	NA
Different criteria	2 (1.1)	1 (1.0)	1 (1.2)		
Not specified	10 (5.6)	4 (4.1)	6 (7.3)		
Index date predefined	46 (25.6)	24 (24.5)	22 (26.8)	.85	0.04
Index date similar to the treatment arm as reported by the authors	39 (84.8)	21 (87.5)	18 (81.8)	.69^c^	NA
Missing data reported when obtaining external control data	13 (7.2)	7 (7.1)	6 (7.3)	>.99	0
Type of missing data					
Baseline data	8 (61.5)	5 (71.4)	3 (50.0)	.08^c^	NA
Outcome data	2 (15.4)	2 (28.6)	0		
Baseline and outcome and treatment data	3 (23.1)	0	3 (50.0)		
Methods of dealing with missing data					
Deletion	7 (53.8)	4 (57.1)	3 (50.0)	>.99^c^	NA
Imputation	6 (46.2)	3 (42.9)	3 (50.0)		

Abbreviations: NA, not applicable; Q1, journals in the top 25% in their Journal Citation Reports category.

^a^ Unless otherwise indicated, calculated using Pearson χ^2^ test.

^b^ Unless otherwise indicated, *df* = 1.

^c^ Calculated using Fisher exact test.

^d^ *df* = 3.

^e^ Defined using the absolute difference in the year of the start of data collection between the treatment group and the external control group.

^f^ Calculated using Mann-Whitney test.

^g^ *df* = 2.

### Methodological Characteristics of Generating External Controls

Table 3 shows the methodological characteristics involved in generating external controls. Of the 180 ECTs, 164 (91.1%) reported important covariates, of which 37 (22.6%) specified the methods used to identify important covariates and 8 (4.9%) were prespecified in the protocol or the statistical analysis plan. Only 60 ECTs (33.3%) used statistical methods to adjust for important covariates when generating the external controls, including the propensity score (PS) (35 [58.3%]); others, such as the disease risk score, used entropy balancing (25 [41.7%]). Of the 35 ECTs that used PS, the most common method was PS weighting (20 [57.1%]), followed by PS matching (8 [22.9%]), PS adjustment (5 [14.3%]), and PS stratification (1 [2.9%]) (eTable 3 in Supplement 1). The different sources of external control data and the use of statistical methods are shown in eFigure 2 in Supplement 1.

**Table 3.** Methodological Characteristics of Generating External Controls

Characteristic	Study group, No. (%)			*P* value^a^	χ^2^ Value^b^
	Total (N = 180)	Q1 journal (n = 98)	Non-Q1 journal (n = 82)		
Important covariates were reported	164 (91.1)	91 (92.9)	73 (89.0)	.52	0.41
Methods of identifying important covariates were reported	37 (22.6)	17 (18.7)	20 (27.4)	.26	1.30
Methods of identifying important covariates					
Review relevant studies and/or combine expertise	36 (97.3)	17 (100)	19 (95.0)		
Based on statistical testing or model	1 (2.7)	0	1 (5.0)		
Important covariates were prespecified in protocol or statistical analysis plan	8 (4.9)	8 (8.8)	0	.009^c^	NA
Statistical methods were used to adjust for important covariates when generating external control	60 (33.3)	31 (31.6)	29 (35.4)		
Propensity score	35 (58.3)	15 (48.4)	20 (69.0)	.71	0.14
Other methods^d^	25 (41.7)	16 (51.6)	9 (31.0)		
Balance of covariates between the treatment and external control arms was checked	138 (76.7)	73 (74.5)	65 (79.3)		
Methods used for checking balance of covariates				.56	0.33
Standardized mean difference	18 (13.0)	7 (9.6)	11 (16.9)		
*P* values from hypothesis testing (eg, *t* test, χ^2^ test)	104 (75.4)	58 (79.5)	46 (70.8)		
Graphical displays	2 (1.4)	1 (1.4)	1 (1.5)		
Only describe covariate comparisons	20 (14.5)	10 (13.7)	10 (15.4)		
Presentation of balance assessment					
Report the balance assessment results in the unmatching groups	80 (44.4)	46 (46.9)	34 (41.5)	.72	1.36^e^
Only report the balance assessment results in the postmatching groups	31 (17.2)	16 (16.3)	15 (18.3)		
Report the balance assessment results in the prematching and postmatching groups	16 (8.9)	6 (6.1)	10 (12.2)		
Not specified	53 (29.4)	30 (30.6)	23 (28.0)		
Any unbalanced covariates reported	71 (55.9)	35 (51.5)	36 (61.0)	.36	0.81
Any unbalanced covariates remained in the postmatching groups (n = 48)	21 (43.8)	8 (33.3)	13 (54.2)	.24	1.35
Any unbalanced covariates exhibited in the unmatching groups (n = 79)	50 (63.3)	27 (61.4)	23 (65.7)	.87	0.03
No. of unbalanced covariates, median (IQR)	2 (1-4)	2 (1-4)	2 (1-4)	.97^f^	NA

Abbreviations: NA, not applicable; Q1, journals in the top 25% in their Journal Citation Reports category.

^a^ Unless otherwise indicated, calculated using the Pearson χ^2^ test.

^b^ Unless otherwise indicated, *df* = 1.

^c^ Calculated using the Fisher exact test.

^d^ Other methods include entropy balancing (1 [1.7%]), exact matching (2 [3.3%]), disease risk score (1 [1.7%]) or individual matching based on several baseline characteristics (21 [35.0%]), and none of these mention propensity scores.

^e^ *df* = 3.

^f^ Calculated using the Mann-Whitney test.

One hundred thirty-eight ECTs (76.7%) checked the balance of covariates between the treatment and external control arms. Of these, the most commonly used methods to check balance were the *P* value (104 [75.4%]) from hypothesis testing (eg, *t* test or χ^2^ test), and only 18 (13.0%) used standardized mean difference. One hundred twenty-seven ECTs (70.6%) reported the results of between-groups balance assessment, of which 71 (55.9%) reported covariate imbalance between groups, with a median number of imbalanced covariates of 2 (IQR, 1-4). Of the 48 ECTs that reported balance assessment results in the postmatching groups, 21 (43.8%) had unbalanced covariates; of the 79 ECTs that did not use matching or other methods to adjust for confounding but reported the balance assessment results, 50 (63.3%) exhibited unbalanced covariates. No significant differences were observed between Q1 and non-Q1 journals, except for the prespecification of covariates (χ^2^_1_ = 7.63; *P* = .006).

### Methodological Characteristics of Statistical Analysis for Estimating Treatment Effects

Of the 180 ECTs, 146 (81.1%) clearly predefined the primary outcome, with 128 (87.7%) comparing the primary outcome between treatment and external control arms. Among 120 ECTs that did not use statistical methods to address confounding when generating external controls, 91 (75.8%) used univariate analysis to estimate treatment effects, and only 18 (15.0%) used multivariable regression analysis; among 60 ECTs that used statistical methods to generate external controls, 39 (65.0%) used univariate analysis and 15 (25.0%) combined it with multivariable regression analysis. Of the 76 ECTs classified as primary analysis and lacking confounding adjustment methods in external control generation, 58 (76.3%) used univariate analysis to estimate treatment effects, and only 13 (17.1%) used multivariable regression analysis (eTable 1 in Supplement 1). Of the 71 ECTs reporting unbalanced covariates, 56 (78.9%) did not adjust for any unbalanced covariates in their primary analysis. No significant differences were observed between Q1 and non-Q1 journals (Table 4).

**Table 4.** Methodological Characteristics of Statistical Analysis for Estimating Treatment Effects

Characteristics	Study group, No. (%)			*P* value^a^	χ^2^ Value^b^
	Total (N = 180)	Q1 journals (n = 98)	Non-Q1 journals (n = 82)		
**Analysis of primary outcome^c^**					
Primary outcome predefined	146 (81.1)	80 (81.6)	66 (80.5)	>.99	0
If yes, the primary outcome was compared between treatment arm and external control	128 (87.7)	67 (83.8)	61 (92.4)	.18	1.78
Statistical methods used for estimating treatment effects					
For ECTs that used statistical methods to address confounding when generating external controls, No./total No. (%)					
Multivariable regression analysis^d^	15/60 (25.0)	11/31 (35.5)	4/29 (13.8)	.11^e^	NA
Univariate analysis	39/60 (65.0)	16/31 (51.6)	23/29 (79.3)		
Other^f^	5/60 (8.3)	3/31 (9.7)	2/29 (6.9)		
Not specified	1/60 (1.7)	1/31 (3.2)	0/29		
For ECTs that did not use statistical methods to address confounding when generating external controls, No./total No. (%)					
Multivariable regression analysis^d^	18/120 (15.0)	9/67 (13.4)	9/53 (17.0)	.44^e^	NA
Univariate analysis	91/120 (75.8)	54/67 (80.6)	37/53 (69.8)		
Other^f^	2/120 (1.7)	1/67 (1.5)	1/53 (1.9)		
Not specified	9/120 (7.5)	3/67 (4.5)	6/53 (11.3)		
For trials with unbalanced covariates, were these covariates included as adjustment factors in the primary analysis?, No./total No. (%)					
All were adjusted	9/71 (12.7)	5/35 (14.3)	4/36 (11.1)	.32^e^	NA
Partial were adjusted	6/71 (8.5)	1/35 (2.9)	5/36 (13.9)		
No	56/71 (78.9)	29/35 (82.9)	27/36 (75)		
Presentation of results					
Adjusted or matched only	48 (26.7)	25 (25.5)	23 (28)	.30	3.67^g^
Unadjusted or unmatched only	103 (57.2)	61 (62.2)	42 (51.2)		
Both	17 (9.4)	6 (6.1)	11 (13.4)		
Not specified	12 (6.7)	6 (6.1)	6 (7.3)		
If both were reported, statistical results were consistent after adjusting or matching covariates	17 (100)	6 (100)	11 (100)		
Conduct of sensitivity analysis					
Sensitivity analysis conducted	32 (17.8)	19 (19.4)	13 (15.9)	.67	0.18
Results of sensitivity analysis were reported	30 (93.8)	18 (94.7)	12 (92.3)	>.99^d^	NA
Was any result of the sensitivity analysis consistent with primary analysis?					
Yes: consistent	22 (68.8)	12 (63.2)	10 (76.9)	.61^d^	NA
No: primary analysis was statistically significant, while at least 1 sensitivity analysis was not statistically significant	6 (18.8)	5 (26.3)	1 (7.7)		
No: primary analysis was not statistically significant, while at least 1 sensitivity analysis was statistically significant	2 (6.3)	1 (5.3)	1 (7.7)		
Not specified	2 (6.3)	1 (5.3)	1 (7.7)		
If no, the reason for inconsistence with the primary analysis was discussed, No./total No. (%)	2/8 (25.0)	1/6 (16.7)	1/2 (50.0)		
**Conduct of QBA**					
QBA conducted	2 (1.1)	0	2 (2.4)		
Rational					
To quantify bias and nullification analysis	1 (50.0)	0	1 (50.0)	NA	NA
Nullification analysis^h^	1 (50.0)	0	1 (50.0)	NA	NA
Use of QBA method	0	0	0	NA	NA
Type of bias modeled					
Uncontrolled confounder	2 (100)	0	2 (100)	NA	NA
Bias parameter source					
External validation (ie, literature/expert opinion)	1 (50.0)	0	1 (50.0)	NA	NA
Not specified	1 (50.0)	0	1 (50.0)	NA	NA
Results of QBA consistent with the primary analysis	2 (100)	0	2 (100)	NA	NA
Effect of bias on the results discussed	2 (100)	0	2 (100)	NA	NA

Abbreviations: ECT, externally controlled trial; NA, not applicable; Q1, journals in the top 25% in their Journal Citation Reports category; QBA, quantitative bias analysis.

^a^ Unless otherwise indicated, calculated using the Pearson χ^2^ test.

^b^ Unless otherwise indicated, *df* = 1.

^c^ We defined a primary outcome for each ECT using predefined criteria. Additionally, if a study prespecified a primary outcome but used a secondary outcome for the comparison between the treatment and external control arm, we selected this secondary outcome as the extraction outcome for assessing subsequent methodological characteristics. We selected statistical analyses of the primary outcome and selected outcome as the primary analysis for extracting methodological characteristics.

^d^ These multivariable analyses were performed using Cox proportional hazards model, Fine-Gray semiparametric proportional hazards regression model, linear regression model, or covariance analysis.

^e^ Calculated using the Fisher exact test.

^f^ Includes mixed-effects model and generalized estimating equation model.

^g^ *df* = 3.

^h^ Bias analysis for an uncontrolled confounder identifies the bias parameter combinations needed to mask a true null association and create the observed effect. This helps determine if an unmeasured confounder could plausibly explain the result.

### Methodological Characteristics of Sensitivity Analysis and Quantitative Bias Analysis

Only 32 ECTs (17.8%) performed a sensitivity analysis on the primary outcome. Of these studies, the most common type of sensitivity analysis was the generation of multiple external control arms (eg, the generation of external controls with different matching methods; 17 [53.1%]) (eTable 4 in Supplement 1). Thirty of these 32 ECTs (93.8%) presented the results of the sensitivity analysis, 8 (25.0%) showed that the results of the sensitivity analysis were inconsistent with the primary analysis, and only 2 of these (25.0%) discussed the reason for the inconsistency. Among 105 ECTs classified as primary analysis, only 14 (13.3%) performed sensitivity analyses (eTable 1 in Supplement 1). No significant differences were observed between Q1 and non-Q1 journals (Table 4).

Only 2 ECTs (1.1%) performed quantitative bias analysis, one to quantify the magnitude and direction of potential biases on the primary outcome and the other as a nullification analysis. Of these 2 ECTs, neither clearly specified the method of quantitative bias analysis; both specified the type of bias modeled as uncontrolled confounder, and 1 provided the source of the bias parameters (Table 4).

## Discussion

In this cross-sectional study, we investigated practices in the design, conduct, and analysis of ECTs published from 2010 to 2023. Our results highlighted several critical issues that need further improvement, including absence of justification for using external controls, failure to prespecify external controls in the protocol, insufficient use of confounding adjustment techniques, inadequate sensitivity analyses, and almost no quantitative bias analysis.

We found that the prespecification of external controls was inadequate. Specifically, only 16.1% of ECTs prespecified the use of external controls in the study protocol, including both the ECTs used in the primary analyses (21 of 105 [20.0%]) and those used in the nonprimary analyses (8 of 75 [10.7%]), raising concerns about potential selection bias, as investigators could selectively choose comparators that favored the desired outcome. Furthermore, 35.6% of ECTs provided justification for using external controls. We further identified suboptimal selection and use of external data sources. Ideally, external controls should be derived from contemporaneous clinical trials addressing similar research questions.^194^ However, 90.0% of ECTs used noncontemporaneous historical data as external controls, potentially introducing temporal confounding from clinical practice trends that undermine comparison validity.^195^ Moreover, most ECTs omitted feasibility assessments of external data sources, which can lead to critical comparability gaps.^196^ Although most ECTs acknowledged the importance of covariate reporting, only 22.6% described how covariates were selected. This nontransparency risks post hoc rationalization, where researchers may select covariates based on observed imbalances rather than those related to the primary outcome based on a priori clinical rationale.^197,198^

Our study showed a critical deficiency in the application of statistical methods to reduce bias during the external control generation, which severely limited the mitigation of bias. Of the few studies using statistical techniques, PS methods predominated. The notable absence of alternative approaches, such as disease risk scores and entropy balancing, suggests a stagnation in the adoption of contemporary causal inference frameworks, as these methods can be particularly useful in settings with rare outcomes or high-dimensional covariate data.^199,200,201^ In addition, balance assessment practices were suboptimal. Although 76.7% of studies assessed covariate balance between treatment and external control arms, hypothesis testing (*P* values) was the predominant technique,^202^ while standardized mean difference—a more appropriate method—was rarely used.^203^ Notably, even among ECTs using matching or adjustment methods, 43.8% retained covariate imbalance, highlighting the inadequacy of current strategies to fully address confounding.^194^

The statistical strategies used to estimate treatment effects in the current ECTs varied, and the analyses methods required significant improvement. Among ECTs without confounding adjustment when generating external controls, 75.8% relied on univariate analysis for treatment effects estimation, which may lead to biased conclusions by ignoring confounders despite methodological simplicity.^204,205^ In contrast, the application of statistical adjustment methods such as multivariate regression was relatively limited, even in ECTs classified as primary analysis (13 of 76 [17.1%]). Despite relevant guidelines recommending baseline adjustment to enhance reliability, especially for imbalanced cohorts requiring multivariate methods,^206,207^ 78.9% of ECTs reporting baseline imbalances omitted these adjustments, indicating insufficient attention to baseline characteristics and methodological rigor.

Our study revealed significant deficiencies in the application and reporting of sensitivity and quantitative bias analyses within ECTs. Sensitivity analysis is crucial for evaluating the reliability of results in ECTs, as they are susceptible to bias due to the absence of randomization.^194,208^ However, only 17.8% of ECTs performed such analyses. More critically, among the ECTs classified as primary analyses, where methodological rigor should be paramount, merely 13.3% conducted sensitivity analyses. The sensitivity analyses focused on the creation of an external control (53.1%), highlighting efforts to address selection bias but neglecting other critical uncertainties, such as unmeasured confounding or missing data. More worryingly, quantitative bias analysis, another useful method for quantifying the impact of bias,^209^ was conducted in only 1.1% of ECTs, indicating an almost complete absence of this critical methodology.

There were substantial methodological deficiencies in ECTs. More than half of ECTs were published in Q1 journals, indicating relatively high levels of interest and attention. Although ECTs in Q1 journals showed better practices than those in non-Q1 journals in several areas, including the provision of rationale, trial registration, protocol availability, and the prespecification of an external control in the protocol, the absolute rates remained extremely low. These findings suggest that suboptimal practices were widespread across all publication tiers, which could potentially undermine the credibility and scientific rigor of ECT evidence. This highlights the urgent need for improvements in future research practices.

### Comparison With Other Studies

A small number of studies have investigated the role of external controls in regulatory and HTA submissions, but their focus has centered on policy frameworks, regulatory acceptance, and evidentiary value.^210,211^ For example, Patel et al^7^ highlighted the practical challenges and opportunities of integrating external controls into HTA evaluations but did not systematically assess the methodological practices. Goring et al^212^ synthesized the characteristics of nonrandomized studies using external controls submitted for regulatory review in the US and Europe, focusing primarily on compliance with regulatory pathways, product labeling, effect size estimation, and approval conditions. These studies focused on addressing the “why” and “when” of using external controls (eg, when RCTs are impractical), yet provided limited practical guidance on the design, conduct, and analysis of ECTs.

In contrast, our study comprehensively examined ECT practices, including external data sources, covariate transparency, statistical methodologies, and the use of sensitivity and quantitative bias analyses, to systematically identify methodological issues. Although the FDA has provided guidance on the design and conduct of ECTs,^12^ it inadequately covers causal inference methods and quantitative bias analysis. Our findings highlight specific deficiencies and provide novel methodological perspectives for improving ECT best practices.

### Implication for Future Research

This study identified critical areas for improvement in ECTs, highlighting the need for methodological enhancements to ensure robust evidence. These include prespecifying external controls to minimize post hoc bias, conducting comprehensive comparability assessments, using causal inference methods to adjust for confounding, and implementing sensitivity analyses to assess the robustness of study results and quantitative bias analyses to evaluate the unavoidable bias impacts. Based on our findings, we propose 13 actionable recommendations for improving the ECT practices, ensuring that these studies generate reliable evidence (Box).^12,213,214,215,216^

Box. **Recommendations on Design, Conduct, and Analysis of Externally Controlled Trials**

**Step 1. Design of the Protocol**

Specify the justification for using external controls in advance, for example in cases of rare diseases where conducting a randomized clinical trial (RCT) is unfeasible.
Prespecify external control selection criteria, data sources, and analytical plans in study protocols to reduce the risk of post hoc bias.

**Step 2. Select External Control Sources**

Consider using external control sources (eg, clinical trial or clinical data) from the same or a similar time period to that of the intervention arm, or as close in time as possible to minimize temporal confounding. If more distant historical data are used, it is crucial to clearly specify whether and how the standard care has evolved over this period.
Predefine key study elements, including covariates, treatments, and outcomes, with emphasis on selecting clinically relevant covariates that significantly influence the primary outcome.
Conduct feasibility assessments of external control data sources to assess the accuracy, completeness, and reliability of the data, while ensuring that an adequate sample size is available for establishing an external control.
Evaluate the comparability of data between the treatment and the external control arm across 10 dimensions in alignment with US Food and Drug Administration guidance, to ensure sufficient similarity. These dimensions include time periods, geographic region, disease diagnostic criteria, prognosis, treatments, other treatment-related factors, follow-up periods, intercurrent events, outcome, and missing data.^12^

**Step 3. Generate External Control**

Screen initial external control samples from the source data using consistent inclusion and exclusion criteria as the experimental group.
Use statistical methods, such as propensity score–based approaches (eg, matching or weighting) when generating external controls. These methods can help adjust for baseline covariates and potential confounding factors. Additionally, appropriate techniques, such as standardized mean differences, should be used to assess the balance of baseline covariates between the treatment and external control arms.^213^

**Step 4. Treatment Effect Estimation**

Adjust for important baseline covariates in the primary analysis to account for potential imbalances in characteristics between the treatment and external control arms.
Use multivariable regression models over univariate analysis to estimate treatment effects when the original external data are used directly as the external control group (ie, generating external controls without statistical methods to address confounding).

**Step 5. Sensitivity and Quantitative Bias Analyses**

Perform sensitivity analyses based on a priori assumptions, such as various methods for adjusting for confounding and for handling missing data, to assess the robustness of the study results.^214^
Conduct quantitative bias analyses, such as E-value, to estimate the magnitude and uncertainty associated with bias in treatment effects.^215,216^

**Step 6. Interpret Results and Draw Conclusions**

Draw well-supported conclusions by conducting a comprehensive interpretation of the results from primary analyses, sensitivity analyses, and quantitative bias analyses, thereby ensuring the transparency of the research.

In addition, given the absence of universally accepted reporting guidelines for ECTs, a collaborative effort involving regulators, researchers, and journal editors is urgently needed to establish reporting standards. Currently, we suggest adopting the STROBE guidelines for ECT reporting, due to their methodological similarity with comparative cohort studies where confounding adjustment is essential. Such standards would promote transparency, reproducibility, and regulatory acceptance of ECTs.

### Strengths and Limitations

This study has several strengths. We used a standardized methodology that included a systematic literature search with predefined criteria, duplicate citation screening, and data abstraction using pilot-tested forms, ensuring reproducibility and minimizing observer variability. Our analysis of 180 ECTs across diverse therapeutic areas, including oncology and rare diseases, represents, to our knowledge, the most comprehensive evaluation in this field, revealing consistent methodological gaps across journals. Importantly, we translated these findings into actionable recommendations to enhance ECT reliability in design, conduct, and analysis. These methodological insights may extend beyond ECTs, offering valuable guidance for improving practices in other nonrandomized intervention studies.

This study also has several limitations. First, systematic searches were confined to PubMed, excluding regulatory databases such as the FDA database and the European Medicines Agency database of European public assessment reports, potentially omitting drug-approval ECTs. Second, our survey findings relied solely on published articles; however, if researchers had followed unpublished protocol details, this could influence our assessment. Third, we focused on 2 ECT designs using individual patient-level data (a single-arm trial with external control and a treatment group from an RCT compared against external control), which limits generalizability to studies using external controls to supplement internal controls or studies using summary-level external controls. Fourth, unadjusted multiple comparisons in Q1 vs non-Q1 journal exploratory analyses may increase type I error risk for *P* values.

## Conclusions

In this cross-sectional study of ECTs, we found that current practices in their design, conduct, and analysis remain suboptimal, which limits their reliability and credibility. We identified critical methodological issues, including the absence of justification for the use of external controls and the failure to prespecify them in the protocols. Other issues included insufficient application of confounding adjustment techniques, inadequate sensitivity analyses, and near absence of quantitative bias analysis. Based on these findings, we proposed 13 recommendations for improving practices. Furthermore, our findings highlighted the urgent need for researchers, regulators, and journal editors to work together to establish standardized methodological and reporting guidelines for ECTs.

## Notes

Supplement 1. **eAppendix.** Search Strategy **eMethods 1.** Data Collection Information **eMethods 2.** Definition of Key Statistical Terms **eFigure 1.** Flowchart of Study Selection **eFigure 2.** Numbers of Included Studies on External Control Data Sources and the Use of Statistical Methods **eTable 1.** Characteristics Between ECTs Classified as Primary Analysis and Nonprimary Analysis (n = 180) **eTable 2.** Characteristics of Sample Size Calculation (n = 180) **eTable 3.** Type of propensity score (n = 35) **eTable 4.** Characteristics of Sensitivity Analysis (n = 32)

Supplement 2.
Data Sharing Statement
