# Supplementary material for: Design, Conduct, and Analysis of Externally Controlled Trials
Source: JAMA Netw Open. 2025 Sep 4;8(9):e2530277. doi: 10.1001/jamanetworkopen.2025.30277 (PMC12411980; doi:10.1001/jamanetworkopen.2025.30277)
Supplement: Supplement 1. — eAppendix. Search Strategy eMethods 1. Data Collection Information eMethods 2. Definition of Key Statistical Terms eFigure 1. Flowchart of Study Selection eFigure 2. Numbers of Included Studies on External Control Data Sources and the Use of Statistical Methods eTable 1. Characteristics Between ECTs Classified as Primary Analysis and Nonprimary Analysis (n = 180) eTable 2. Characteristics of Sample Size Calculation (n = 180) eTable 3. Type of propensity score (n = 35) eTable 4. Characteristics of Sensitivity Analysis (n = 32) [file jamanetwopen-e2530277-s001.pdf]

Supplementary Online Content

Lui J, Yao M, Wang M, et al. Design, conduct, and analysis of externally controlled trials. *JAMA Netw Open.* 2025;8(9):e2530277.
doi:10.1001/jamanetworkopen.2025.30277

eAppendix. Search Strategy

eMethods 1. Data Collection Information

eMethods 2. Definition of Key Statistical Terms

eFigure 1. Flowchart of Study Selection

eFigure 2. Numbers of Included Studies on External Control Data Sources and the Use of Statistical Methods

eTable 1. Characteristics Between ECTs Classified as Primary Analysis and Nonprimary Analysis (n = 180)

eTable 2. Characteristics of Sample Size Calculation (n = 180)

eTable 3. Type of Propensity Score (n = 35)

eTable 4. Characteristics of Sensitivity Analysis (n = 32)

This supplementary material has been provided by the authors to give readers additional information about their work.

© 2025 Lui J et al. *JAMA Network Open*.

eAppendix 1. Search strategy

PubMed

#1 external control [mh] OR external control* [tw] OR external compar* [tw]

#2 synthetic control [mh] OR synthetic control* [tw] OR synthetic compar* [tw]

#3 historical control [mh] OR historical control* [tw] OR historical compar* [tw]

#4 natural history control [mh] OR natural history control* [tw] OR natural history compar* [tw]

#5 contemporaneous control [mh] OR contemporaneous control* [tw] OR contemporaneous compar* [tw]

#6 non-contemporaneous control [mh] OR non-contemporaneous control* [tw] OR non-contemporaneous compar* [tw]

#7 OR/#1-6

#8 single arm [mh] OR single arm [tw] OR clinical trial [pt]

#9 #7 AND #8

#10 animals [mh] NOT Humans [mh]

#11 #9 NOT #10

#12 "2010/01/01"[dp] : "2023/12/31"[dp]

#13 #11 AND #12

© 2025 Lui J et al. *JAMA Network Open*.

eMethods 1. Data Collection Information

General characteristics: Journal ranks by quartile (Q), author, year of publication, study type, disease area, treatment type, number of external controls, type of external controls, reason for using external control, trial registration, trial protocol available, pre-specified for using external control, funding sources, conflict of interest disclosure.

We also documented the characteristics of sample size calculation, including sample size, whether a sample size calculation was performed, whether the sample size calculation was based on primary outcome, significance level specified, one-sided or two-sided test, power specified, sample size adjusted appropriately for specific situation, sample size changed during the trial.

We documented the reported analysis types of ECTs. We classified each ECT analysis type as either "primary analysis" or "non-primary analysis" according to the following strategy. For a first-reported single-arm trial, if it had a pre-specified external control group in the protocol and used a pre-specified primary outcome to estimate the treatment effect between the treatment and the external control arm, or if it lacked a pre-specified external control group but still used a pre-specified primary outcome for such an estimation, the analysis type was defined as "primary analysis". Conversely, if a first-reported single-arm trial had a pre-specified primary outcome but used a secondary outcome to estimate the treatment effect between the treatment and the external control arm, the analysis type was classified as "non-primary analysis". Moreover, if a study was based on a previously published single-arm trial and selected an external control group post hoc for treatment effect estimation, or used one group from a RCT along with an external control group for effect estimation analysis, the analysis type was also classified as "non-primary analysis". Finally, if a study explicitly reported itself as an exploratory or post hoc or other non-primary analysis, the analysis type was classified as "non-primary analysis".

Characteristics of external control data sources: We documented the sources of external control data; the periods of external data collection, the difference in data collection time between the treatment arm and external control arm; whether external control data were pre-assessed, including assessment of accuracy and completeness; whether patient diagnostic, inclusion and exclusion criteria were the same or similar to those of the treatment arm; whether treatment characteristics of the external control arm

© 2025 Lui J et al. *JAMA Network Open*.

were reported, whether combined interventions and adherence of the external control arm were reported; whether the same criteria were used to define outcome; whether the index date was predefined, whether the index date was similar to that of the treatment arm; whether any data were not be available (i.e., missing data) when obtaining external control data, type of missing data, methods used to deal with missing data.

We used following criteria to assess the similarity of outcome definitions. Definitions were considered "same or similar" if: authors explicitly stated same/similar criteria, or unspecified in text but investigator assessment confirmed that 1) measurement tools, thresholds, and timeframes matched exactly; 2) for objective endpoints (e.g., all-cause mortality, organ transplant, unplanned hospitalization), identical operational definitions were presumed. Definitions were considered "different" if: authors explicitly stated the outcome definitions of treatment and external control groups, but there was at least one discrepancy in definition components (e.g., differing BMI thresholds for obesity-related outcomes), or if non-identical measurement instruments/versions.

Methodological characteristics of generating external control: We documented whether important covariates (e.g., sex, age, severity, comorbidities, prior and current treatments received) were reported and prespecified, methods used to identify important covariates, whether any statistical method (e.g., propensity score (PS), or other matching methods) was used to adjust for important covariates, statistical methods, type of PS, whether balance of covariates between groups was checked, methods used to check the balance of covariates, whether the results of the assessment of balance between groups were reported, presentation of the results of the assessment of balance, whether any unbalanced covariate was reported (any unbalanced covariates were reported in the post-matching groups and un-matching groups), number of unbalanced covariates.

Since the included studies did not explicitly state that the reported covariates were those associated with the outcome, we assumed that all imbalance covariates reported in the studies were key variables that may be related to the outcome.

Methodological characteristics of statistical analysis for estimating treatment effects: We defined a primary outcome for each ECT using the following strategy: if a trial prespecified a primary outcome for the comparison between the treatment arm and the

© 2025 Lui J et al. *JAMA Network Open*.

external control arm, we selected it as the primary outcome for our analyses; if a trial prespecified multiple primary outcomes, we selected the first one mentioned in the methods; if a trial did not to specify a primary outcome in the methods, we selected the first reported outcome in the results. In addition, if a study prespecified a primary outcome but used a secondary outcome for the comparison between the treatment and the external control arm, we selected this secondary outcome as the extraction outcome for assessing subsequent methodological characteristics.

We documented the characteristics of the primary outcome, including whether a primary outcome was predefined in the methods. We documented the statistical analysis of the primary outcome or selected outcome, including statistical methods used for estimating treatment effects, whether unbalanced covariates were included as adjustment factors in the primary analysis of trials with unbalanced covariates, presentation of the results (adjusted or matched only, unadjusted or unmatched only, both, not specified), if both were reported, and whether statistical results were consistent after adjustment or matching for covariates.

Methodological characteristics of sensitivity analysis: We documented whether any sensitivity analysis was performed on the primary outcome or selected outcome, number of sensitivity analyses, type of sensitivity analysis, whether the results of the sensitivity analysis were reported, whether any result of the sensitivity analysis was consistent with the primary analysis (yes, no, not specified), if no, and whether the reason for the inconsistence with the primary analysis was discussed.

Methodological characteristics of quantitative bias analysis: We documented whether a quantitative bias analysis was performed, rational for the quantitative bias analysis, method of the quantitative bias analysis (e.g., E-value), type of bias modelled, source of the bias parameters, whether the results of quantitative bias analysis were consistent with the primary analysis, and whether the impact of bias on the results was discussed.

eMethods 2. Definition of Key Statistical Terms

Bias: Systematic deviation of results from true effects due to flaws in design, measurement, or analysis (e.g., confounding in non-randomized studies).

Propensity Scores (PS): it refers to the conditional probability of a unit (e.g., person,

© 2025 Lui J et al. *JAMA Network Open*.

classroom, school) being assigned to the treatment, given a set of observed covariates.

Generating external controls: it refers to the process of generating external control samples from raw external control source data (e.g., real-word data or trial data).

E-value: The E-value is the minimum strength of association on the risk ratio scale that an unmeasured confounder would need to have with both the treatment and the outcome, conditional on the measured covariates, to explain away a treatment-outcome association.

Matching methods: it refers to reduce bias for the estimated treatment effect in an observational-data study or quasi-experiment (i.e. when the treatment is not randomly assigned), by finding, for every treated unit, one (or more) non-treated unit(s) with similar observable characteristics against which the covariates are balanced out.

© 2025 Lui J et al. *JAMA Network Open*.

eFigure 1. Flowchart of Study Selection

One article reported three ECTs; therefore, we included a total of 180 ECTs from 178 articles.

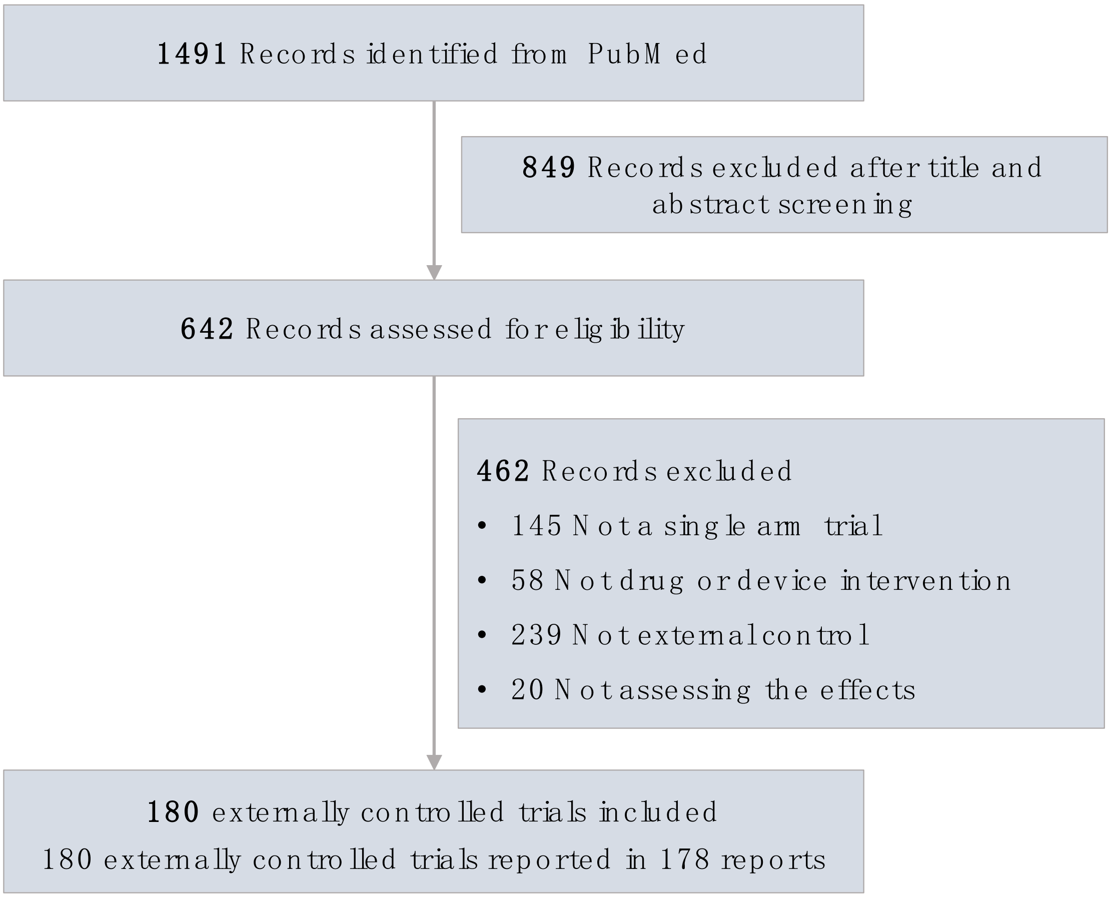

© 2025 Lui J et al. *JAMA Network Open*.

eFigure 2. Numbers of Included Studies on External Control Data Sources and the Use of Statistical Methods

Some studies derived external data from multiple sources, such as an external control group for a study derived from both EMRs and published trials. Other methods include entropy balancing, coarsened exact matching, disease risk score, or individual matching based on several baseline characteristics and none of these mention propensity scores.

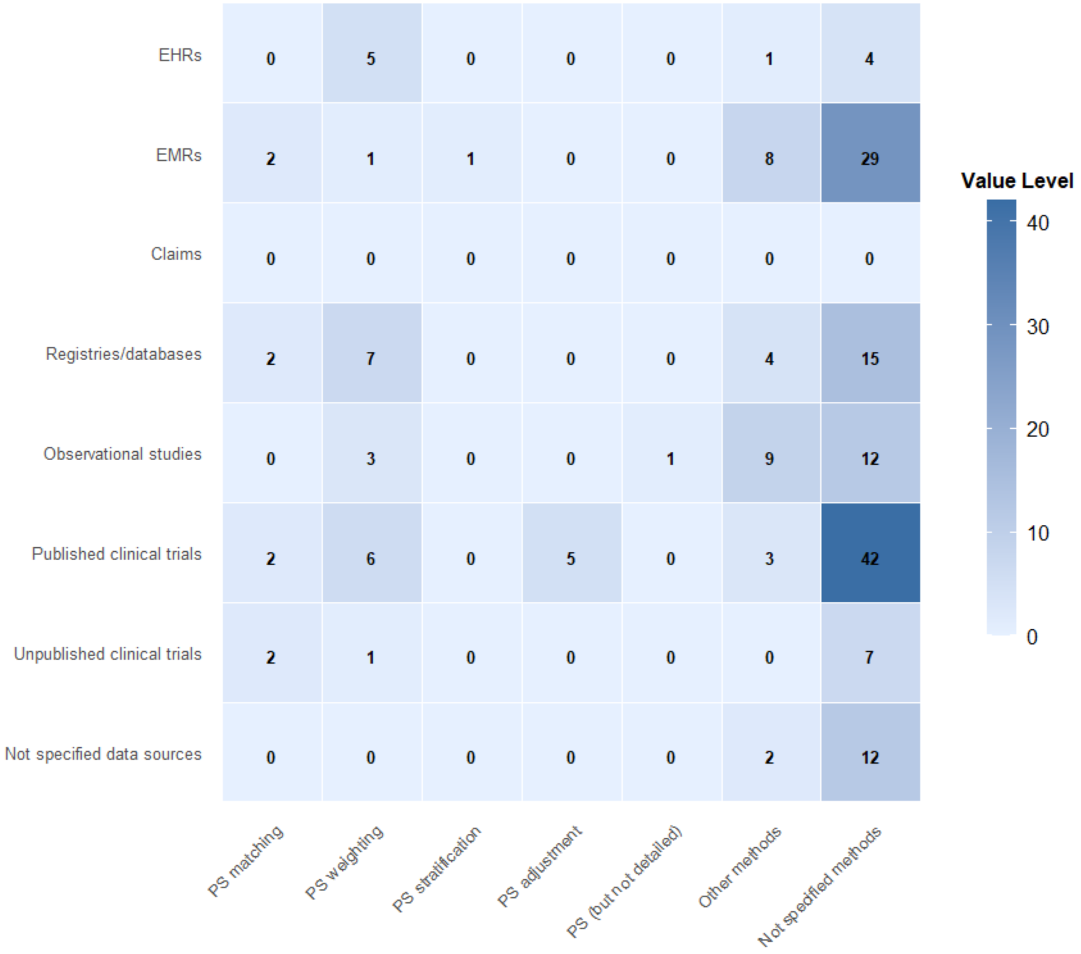

© 2025 Lui J et al. *JAMA Network Open*.

eTable 1. Characteristics Between ECTs Classified as Primary Analysis and Nonprimary Analysis (n = 180)

Characteristics	Primary analyses (N=105, %)	non-primary analyses (75, %)	P	χ^2
**Type of study**			<0.001	11.69
Single arm trial vs. external control	103 (98.1)	62 (82.7)		
An arm derived from RCT vs. external control	2 (1.9)	13 (17.3)		
**Predesigned for using external control the study protocol: Yes**	21 (20)	8 (10.7)	0.141	2.17
**Sources of external control data**			0.733[a]	
Real world data	56 (53.3)	42 (56)		
Trial data	40 (38.1)	27 (36)		
Observational and Trial data	0 (0)	1 (1.3)		
Not specified	9 (8.6)	5 (6.7)		
**Matching method was used to adjust for important covariates when generating external control: Yes**	29 (27.6)	31 (41.3)	0.077	3.11
Propensity score	17 (58.6)	18 (58.1)		
Other matching methods	12 (41.4)	13 (41.9)		
**Statistical methods used for estimating treatment effects**			0.095[a]	
For ECTs that used statistical methods to address confounding when generating external controls (n=60)	29	31	0.235[a]	
Multivariate analysis [b]	6 (20.7)	9 (29)		
Univariate analysis	22 (75.9)	17 (54.8)		
Other [c]	1 (3.4)	4 (13)		
Not specified	0 (0)	1 (3.2)		
For ECTs that did not use statistical methods to address confounding when generating external controls (n=120)	76	44	0.162[a]	
Multivariate analysis [b]	13 (17.1)	5 (11.4)		
Univariate analysis	58 (76.3)	33 (75)		
Other [c]	2 (2.6)	0 (0)		
Not specified	3 (4)	6 (13.6)		
**Sensitivity analysis conducted: Yes**	14 (13.3)	18 (24)	0.099	2.72
**Quantitative bias analysis conducted: Yes**	0 (0)	2 (2.7)		

[a] Fisher's exact test; others are Pearson's chi-squared test

[b] These Multivariable analyses were performed using Cox proportional hazards model, Fine Gray semiparametric proportional hazards regression model, linear regression model, or covariance analysis.

[c] Other include mixed-effects model and generalized estimating equation model.

© 2025 Lui J et al. *JAMA Network Open*.

eTable 2. Characteristics of Sample Size Calculation (n = 180)

**Characteristics**	**Total (n=180, %)**	**Q1 journal (n=98, %)**	**Non-Q1 journal (n=82, %)**	**P**	χ^2
**Sample size** [median (IQR)]					
Treatment arm	47 (25-101)	46 (25-100)	56 (25-101)	0.89[b]	
External control arm (raw/ unadjusted/ unmatching)	83 (37-159)	75 (34-145)	99 (37-171)	0.72[b]	
External control arm (adjusted/ matching)	89 (44-146)	88 (46-142)	91 (44-158)	0.908[b]	
**Sample size calculated**	56 (31.1)	31 (31.6)	25 (30.5)	0.774[a]	
Calculate the sample size for the treatment arm	37 (20.6)	22 (22.4)	15 (18.3)		
Calculate the sample size for the external control arm	3 (1.7)	1 (1)	2 (2.4)		
Calculate the sample size for the treatment and external control arm	16 (8.9)	8 (8.2)	8 (9.8)		
No	124 (68.9)	67 (68.4)	57 (69.5)		
**Sample size calculated based on primary outcome (n=56)**				0.502[a]	
Yes	53 (94.6)	28 (90.3)	25 (100)		
No	3 (5.4)	3 (9.7)	0 (0)		
**Significance level (α) specified (n=56)**				0.454	0.56
Yes	44 (78.6)	26 (83.9)	18 (72)		
No	12 (21.4)	5 (16.1)	7 (28)		
**α** [median (range)]	0.05 (0.01-0.2)	0.05 (0.01-0.2)	0.05 (0.025-0.1)		
**One-sided or two-sided test (n=56)**				0.949	0.10[c]
One-side test	25 (44.6)	14 (45.2)	11 (44)		
Two-sided test	19 (33.9)	10 (32.3)	9 (36)		
Not specified	12 (21.4)	7 (22.6)	5 (20)		
**Power (1 - β) specified (n=56)**				1[a]	
Yes	48 (85.7)	27 (87.1)	21 (84)		
No	8 (14.3)	4 (12.9)	4 (16)		
**1 – β** [median (range)]	0.8 (0.8-0.9)	0.8 (0.8-0.9)	0.8 (0.8-0.9)		
**Sample size adjusted appropriately by specific situations** [d] **(n=56)**				0.115[a]	

© 2025 Lui J et al. *JAMA Network Open*.

Yes, adjusted by loss to follow-up	18 (32.1)	7 (22.6)	11 (44)	
Yes, adjusted by other situations	2 (3.6)	2 (6.5)	0 (0)	
No	36 (64.3)	22 (71)	14 (56)	
If adjusted by loss to follow-up, the rate of loss to follow-up (median range)	0.15 (0.01-0.3)	0.1 (0.01-0.2)	0.2 (0.08-0.3)	
**Sample size modified during the trial (n=56)**				0.398[a]
Yes, increased the sample size	8 (14.3)	3 (9.7)	5 (20)	
Yes, decreased the sample size	2 (3.6)	2 (6.5)	0 (0)	
No	46 (82.1)	26 (83.9)	20 (80)	

[a] Fisher's exact test; others are Pearson's chi-squared test

[b] Values are median (IQR) and P value are from Mann-Whitney U test; IQR, interquartile range

[c] The degree of freedom of this characteristic is 2, and the other degrees of freedom are 1.

[d] Specific situations refer to adjustments in the sample size made to account for potential loss to follow-up (e.g., by increasing the sample size), or other situations such as increasing the sample size in anticipation of conducting subgroup analyses.

eTable 3. Type of Propensity Score (n = 35)

Characteristics	Total (n=35, %)	Q1 journals (n=15, %)	Non-Q1 journals (n=20, %)	P
**Type of PS**				0.054[a]
PS matching	8 (22.9)	5 (33.3)	3 (15)	
PS weighting	20 (57.1)	8 (53.3)	12 (60)	
PS stratification	1 (2.9)	1 (6.7)	0 (0)	
PS adjustment	5 (14.3)	0 (0)	5 (25)	
Not specified	1 (2.9)	1 (6.7)	0 (0)	

[a] Fisher's exact test

eTable 4. Characteristics of Sensitivity Analysis (n = 32)

Characteristics	Total (n=32, %)	Q1 journals (n=19, %)	Non-Q1 journals (n=13, %)	P
**Number of sensitivity analysis**				1[a]
1	12 (37.5)	7 (36.8)	5 (38.5)	
> 1	20 (62.5)	12 (63.2)	8 (61.5)	
**Type of sensitivity analysis**				
Generating external control arm	17 (53.1)	9 (47.4)	8 (61.5)	
Dealing with missing data	3 (9.4)	2 (10.5)	1 (7.7)	

© 2025 Lui J et al. *JAMA Network Open*.

Adjustment of covariates	5 (15.6)	2 (10.5)	3 (23.1)
Comparing different statistical models	4 (12.5)	3 (15.8)	1 (7.7)
Comparing multiple datasets (e.g., ITT analysis, PP analysis	3 (9.4)	2 (10.5)	1 (7.7)
Other (e.g., different disease status)	8 (25)	5 (26.3)	3 (23.1)

[a] Fisher's exact test.

© 2025 Lui J et al. *JAMA Network Open*.
