# Supplementary material for: Design, Conduct, and Analysis of Externally Controlled Trials
Source: JAMA Netw Open. 2025 Sep 4;8(9):e2530277. doi: 10.1001/jamanetworkopen.2025.30277 (PMC12411980; doi:10.1001/jamanetworkopen.2025.30277)
Supplement: Supplement 2. — Data Sharing Statement [file jamanetwopen-e2530277-s002.pdf]

Data Sharing Statement

Liu. Design, Conduct, and Analysis of Externally Controlled Trials. *JAMA Netw Open*.
Published September 04, 2025. doi:10.1001/jamanetworkopen.2025.30277

Data
Data available: Yes
Data types: Data (not involving human participants)
How to access data: All data generated and analyzed for this study are available in the article or supplementary material.
When available: With publication

Supporting Documents
Document types: None

Additional Information
Who can access the data: Any academic researcher requesting the data
Types of analyses: For research purpose
Mechanisms of data availability: After approval of a proposal
